# Evaluating HPV Vaccination‐Related Content on a Burgeoning Social Media Platform: Insufficient Quality of TikTok

**DOI:** 10.1002/oto2.70052

**Published:** 2024-12-15

**Authors:** Matthew E. Lin, Oluwatobiloba Ayo‐Ajibola, Carlos X. Castellanos, Jonathan D. West, Neil Luu, Niels C. Kokot

**Affiliations:** ^1^ Department of Head and Neck Surgery David Geffen School of Medicine at University of California Los Angeles Los Angeles California USA; ^2^ Keck School of Medicine of the University of Southern California Los Angeles California USA; ^3^ Caruso Department of Otolaryngology–Head and Neck Surgery Keck School of Medicine of the University of Southern California Los Angeles California USA

**Keywords:** head and neck cancer, HPV, quality, reliability, social media, TikTok, vaccination

## Abstract

**Objective:**

Assessing the quality of human papillomavirus (HPV) vaccination‐related content on TikTok is crucial due to its popularity among adolescents. We assessed these videos while comparing the content and quality of videos with and without physician involvement.

**Study Design:**

Cross‐sectional cohort analysis.

**Setting:**

HPV vaccination‐related TikTok videos.

**Methods:**

The TikTok library was queried using the search terms #HPVvaccine, #HPVvaccination, #Gardasil, #Gardasilvaccine, and #Gardasilvaccination. Video quality was evaluated using the DISCERN scale, assessing treatment‐related information quality. Descriptive statistics were used to characterize our cohort. *t* Test and Fischer's exact test were used to assess for differences in video content and quality based on physician involvement. Significance was set at *P* < .05.

**Results:**

Our search yielded 131 videos, averaging 68,503.12 views, 2314.27 likes, and 89.28 comments per video. Videos frequently involved physicians (48.09%), focused on education (54.96%) or advocacy (22.90%), and were US‐made (68.90%). Otolaryngologists were rarely featured (3.17%). While most videos mentioned the HPV vaccine protected against cancer generally (86.26%), and cervical cancer specifically (67.94%), few discussed its protective effect against head and neck cancer (26.72%). Videos infrequently discussed updated eligibility among all adults ≤45 years of age (26.72%) or that men can also receive the vaccine (28.24%). Physician‐involved videos were more focused on education (*P* < .001) and focused less on patient experiences (*P* < .001) and advocacy (*P* = .036). Overall DISCERN scores were low among physician (mean = 2.46, SD = 1.13) and nonphysician (mean = 2.09, SD = 1.02) content.

**Conclusion:**

TikTok HPV vaccination content is poor in quality, even with physician involvement. Enhancing content quality and increasing otolaryngologist participation can boost HPV awareness and vaccination rates.

Human papillomavirus‐positive oropharyngeal squamous cell carcinoma (HPV+ OPSCC) is the most common type of head and neck cancer (HNC) in the United States, accounting for 50% to 90% of all OPSCC cases.^1^, ^2^, ^3^ HPV+ OPSCC presents a distinct etiology from its HPV‐negative counterparts and primarily affects a younger, more sexually active population with fewer comorbidities.^4^, ^5^, ^6^ Given the morbidity and mortality associated with HPV‐positive malignancies,^7^, ^8^, ^9^, ^10^ the recent development of an HPV vaccine against high‐risk strains has led to a reduction in the prevalence of HPV and the incidence of HPV‐related cancers.^11^ The Gardasil vaccine, known for its high effectiveness, was approved by the US Food and Drug Association in 2009 and initially recommended for female patients ages 9 to 26.^12^ However, guidelines for HPV vaccination were expanded in 2019 to include both male and female unvaccinated adults up to the age of 45 in an effort to further protect against HPV‐related cancers and diseases.^13^ Moreover, in 2020, vaccination was approved for primary HPV+ OPSCC prophylaxis.^14^

Unfortunately, patients may not be fully aware of the vaccine's effectiveness or current vaccination guidelines, as a lack of knowledge about the vaccine has been previously identified as a barrier to vaccination.^15^ Other studies have found a majority of US adults were unaware of the association between HPV and oropharyngeal, anal, and penile cancers and that a significant portion of young adults aged 18 to 26 were unaware of the efficacy of the HPV vaccine in preventing cervical cancer.^16^, ^17^ This lack of knowledge is particularly concerning given the high rates of sexually transmitted HPV and sexual activity among this age group.^1^, ^18^, ^20^ Vaccine acceptance is also low among males relative to females, likely due to the vaccine being primarily marketed as protective against cervical cancer.^21^, ^22^, ^23^, ^24^ Targeted patient education about HPV is needed, particularly among adolescents and young adults who have a high prevalence of infection but may be less likely to seek out information about the virus or its risks through primary scientific literature.

Social media has seen a meteoric rise in the American zeitgeist, with use among adults rising from 5% in 2005 to 72% in 2021.^25^ Young adults engage with social media much more than previous generations with the platform TikTok arising as a particularly popular option for receiving and disseminating health information.^26^, ^27^, ^28^, ^29^ With over 3 billion downloads, an overall US use prevalence rising from 22% to 33% between 2020 and 2022, and over 67% of its users aged 13 to 24, TikTok is a desirable vector for advising age‐eligible patients on HPV vaccination.^6^, ^25^, ^26^, ^30^ US teenagers, an age group comprising a prime vaccination demographic, the use of social media via smartphones is exceptionally high (95%) compared to young adults (67%) with many citing information gathering, including that relating to health, as the second most popular reason for social media having a positive impact on them.^31^, ^32^

Previous research has analyzed aspects of HPV vaccination information on TikTok, but none have thoroughly evaluated the educational quality of the available content nor have they assessed the prevalence of messaging regarding male and adult vaccine eligibility and the vaccine's protective effect against HPV+ OPSCC.^33^, ^34^ Thus, we aim to evaluate the quality of HPV‐vaccine information on TikTok and determine its relevance to HPV+ OPSCC to better inform efforts to decrease the incidence of this disease.

## Methods

This study was exempt from review by the Institutional Review Board of the University of Southern California due to a lack of patient involvement or personal identifying health information. This was a cross‐sectional analysis of videos related to HPV vaccination posted on TikTok. All TikTok videos about HPV vaccination were anonymously queried in November 2022 on an incognito web browser to avoid account‐specific search biases. The following search terms were used: #HPV, #HPV vaccine, #HPV vaccination, #GardasilVaccine, #Gardasilvaccination.

Video metrics were collected, such as video length, age of video in months, age of posting account in months, and the number of likes, views, comments, and account subscribers. Videos were also classified by type of user account (patient, physician, other health care provider, health care group, health information, entertainment, other), type of video category (patient experience, education, advocacy, self‐promotion, entertainment, other), country of origin (United States and outside United States), and physician specialty (otolaryngology, obstetrician‐gynecologists [OBGYN], family medicine, other) when relevant. Health care groups were defined as clinics, physician groups, and other collections of health care professionals who provide clinical care. Health information accounts were defined as those that specifically educate on health‐related topics, and entertainment accounts were defined as accounts that publish non‐educational content. Educational videos were defined as those created for informative purposes. Patient experience videos were defined as those centered around patients emphasizing the process of becoming vaccinated against HPV. Advocacy videos emphasized the importance of HPV vaccination, self‐promotion videos advertised some sort of medical service or opportunity, and entertainment videos were defined as those that were primarily noneducational in nature.

The DISCERN scale—a 16‐question, 5‐point validated scale that assesses treatment‐related information quality where higher scores indicate higher quality content—was used to assess the informational quality of HPV vaccination‐related videos on TikTok. This scale has been extensively used to evaluate the quality of other treatment‐related online videos and splits its 15 questions into 3 categories: reliability, quality, and overall rating. Scores 4.5 and higher are considered excellent, those from 4.2 to 4.4 are considered very good, 3.4 to 4.1 good, 2.6 to 3.3 average, 1.9 to 2.5 poor, and less than 1.8 very poor. Three reviewers (M.E.L, O.A.A, C.X.C) analyzed a subset of videos together based on DISCERN handbook guidelines to limit inter‐rater variability.

Data analysis was performed in Microsoft Excel and in R. Descriptive statistics were used to characterize our cohort; frequencies and percentages were used for categorical variables and means, and standard deviations were used for continuous variables. Welch's *t* test and Fischer's exact test were used to assess for differences in video content and quality based on physician involvement. Significance was set at *P* < .05.

## Results

### Overall Cohort Characteristics

There were 131 videos analyzed (Table 1), with an average of 68,503.12 views (SD = 137,039.05), 2314.27 likes (SD = 6022.34), 89.28 comments per video (SD = 174.62), and 195,639.94 account subscribers (SD = 413,956.07). The majority of videos originated from the United States (68.90%, n = 90). The most common user account types were categorized as physicians (41.98%, n = 55), patients (19.08%, n = 25), and entertainment (9.92%, n = 3). The most common video categorizations were education (54.96%, n = 72), advocacy (22.90%, n = 30), and patient experience (8.11%, n = 11).

**Table 1:** Overall Video Characteristics

Characteristic	All videos (n = 131) (n, %)
Video metrics (mean, SD)	
Duration, minute	0.75 (0.73)
Likes (#)	2314.27 (6022.34)
Views (#)	68,503.12 (137,039.05)
Comments (#)	89.28 (174.62)
Account subscribers (#)	195,639.94 (413,956.07)
Type of user account	
Patient	25 (19.08)
Physician	55 (41.98)
Other provider	10 (7.63)
Health care group	12 (9.16)
Health Information	7 (5.34)
Entertainment	13 (9.92)
Other	9 (6.97)
Type of video	
Patient experience	11 (8.40)
Education	72 (54.96)
Advocacy	30 (22.90)
Self‐promotion	8 (6.11)
Entertainment	1 (0.76)
Other	8 (6.11)
Country of origin	
United States	90 (68.90)
Non‐United States	41 (31.30)
Specialty	
Otolaryngology	2 (1.53)
OBGYN	22 (16.79)
Family medicine	18 (13.74)
Other	21 (16.03)
Mentioned can vaccinate up to 45	35 (26.72)
Mentioned protects against cancer	113 (86.26)
Cervical cancer	89 (67.94)
Vulva/vaginal cancer	15 (11.45)
Penile cancer	19 (14.50)
Anal cancer	30 (22.90)
Head and neck cancer	35 (26.72)
Cancer, not otherwise specified	14 (10.69)
Mentioned men can receive vaccine	37 (28.24)
DISCERN rating (mean, SD)	
Reliability	2.49 (0.52)
Quality	1.82 (0.49)
Overall	2.27 (1.09)

Abbreviation: OBGYN, obstetrician‐gynecologist.

When analyzed for the inclusion of various vaccination content, videos infrequently discussed updated vaccine eligibility including all adults ≤ 45 years of age (26.72%, n = 35). One hundred thirteen (86.26%) videos mentioned cancer. Videos advocated protection against various cancers to varying degrees; cervical cancer (67.94%, n = 89) was most common, followed by HNC (26.72%, n = 35), anal cancer (22.90%, n = 30), penile cancer (14.50%, n = 19), and vulva/vaginal cancer (11.45%, n = 15). 28.24% of videos mentioned men are eligible to receive the vaccine. The overall cohort received low scores on the DISCERN rating system, exhibiting lower mean reliability (2.49, SD = 0.52), quality (1.82, SD = 0.49), and overall ratings (2.27, SD = 1.09).

### Differences in Video Characteristics Based on Physician Involvement

When stratified for the presence of a physician in the video (Table 2), 63 videos were identified (48.09%, n = 63). Otolaryngologists were rarely featured (3.17%, n = 2), eclipsed by gynecologists (34.92%, n = 22) and primary care physicians (28.57%, n = 18). Compared to videos not featuring a physician (72,240.46, SD = 19,586.8), videos with physician content were posted on accounts with significantly higher mean subscriber counts (328,833.03 vs 7240.46, *P* < .001). Physician videos were significantly more focused on education (80.95% vs 30.88%, *P* < .001) and significantly less focused on patient experiences (0% vs 16.18%, *P* < .001) and advocacy (14.29% vs 30.88%, *P* = .036) compared to nonphysician videos. Physician videos were also less frequently posted on accounts of a miscellaneous nature (0% vs 11.76%, *P* < .001). Physician videos had a significantly higher DISCERN reliability (2.63 vs 2.35, *P* = .002) and quality (1.86 vs 1.78, *P* = .034) score relative to as compared to nonphysician videos; overall DISCERN scores among physician videos were not statistically significant (1.46 vs 2.09, *P* = .051). When comparing the informational content of physician and nonphysician videos, no significant differences were found between the subgroups regarding mentions of cancer, different types of HPV‐related cancers, vaccination eligibility up to 45 years old, and male vaccine eligibility.

**Table 2:** Video Characteristics Stratified by Videos With Physicians and Those Without

Characteristic	Physician (n = 63) (n, %)	Non‐Physician (n = 68) (n, %)	*P* value
Video metrics (mean, SD)			
Duration, minute	0.74 (0.77)	0.76 (0.69)	.840
Likes (#)	2100.08 (4,368.35)	2512.71 (7254.67)	.692
Views (#)	66,492.81 (109,055.11)	70,365.62 (15,9453.53)	.871
Comments (#)	82.7 (134.48)	95.38 (205.79)	.675
Account subscribers (#)	328,833.03 (532,087.59)	72,240.46 (19,586.8)	<.001
Type of user account			
Patient	0 (0.00)	25 (36.76)	<.001
Physician	55 (87.30)	0 (0.00)	<.001
Other providers	0 (0.00)	10 (14.71)	.001
Health care group	6 (9.52)	6 (8.82)	1.000
Health Information	1 (1.59)	6 (8.82)	.117
Entertainment	0 (0.00)	13 (19.12)	<.001
Other	1 (1.59)	8 (11.76)	.034
Type of video			
Patient experience	0 (0.00)	11 (16.18)	<.001
Education	51 (80.95)	21 (30.88)	<.001
Advocacy	9 (14.29)	21 (30.88)	.036
Self‐promotion	2 (3.17)	6 (8.82)	.277
Entertainment	0 (0.00)	1 (1.47)	1.000
Other	0 (0.00)	8 (11.76)	.007
Country of origin			
United States	43 (68.25)	47 (69.12)	1.000
Non‐United States	20 (31.75)	21 (30.88)	1.000
Specialty			
Otolaryngology	2 (3.17)	‐	‐
OBGYN	22 (34.92)	‐	‐
Family medicine	18 (28.57)	‐	‐
Other	21 (33.33)	‐	‐
Mentioned can vaccinate up to 45	20 (31.75)	15 (22.06)	.240
Mentioned protects against cancer	52 (82.54)	61 (89.71)	.311
Cervical cancer	39 (61.90)	50 (73.53)	.191
Vulva/vaginal cancer	7 (11.11)	8 (11.76)	1.000
Penile cancer	9 (14.29)	10 (14.71)	1.000
Anal cancer	18 (28.57)	12 (17.65)	.151
Head and neck cancer	21 (33.33)	14 (20.59)	.116
Cancer, not otherwise specified	4 (6.35)	10 (14.71)	.160
Mentioned men can receive vaccine	21 (33.33)	16 (23.53)	.247
DISCERN rating (mean, SD)			
Reliability	2.63 (0.49)	2.35 (0.51)	.002
Quality	1.86 (0.57)	1.78 (0.41)	.034
Overall	2.46 (1.13)	2.09 (1.02)	.051

Abbreviation: OBGYN, obstetrician‐gynecologist.

### Differences in Video Characteristics Based on Mention of HNC

As seen in Table 3, 35 videos mentioned HNC (26.71%) and 96 did not (73.29%). Videos mentioning HNC were significantly longer than those who did not (1.09 vs 0.63 minutes, *P* = .007) and significantly less likely to be focused on advocacy (8.57% vs 28.13%, *P* = .020). Videos that mentioned HNC had statistically higher DISCERN reliability (2.71 vs 2.41, *P* = .001), quality (2.13 vs 1.71, *P* < .001), and overall scores (2.91 vs 2.03, *P* < .001). Videos mentioning HNC were also significantly more likely to mention cervical cancer (85.71% vs 60.82%, *P* = .007), vulva/vaginal cancer (28/57% vs 5.21%, *P* = .001), penile cancer (40.00% vs 5.21%, *P* < .001), and anal cancer (57.14% vs 10.31%, *P* < .001). There were no significant differences between videos mentioning HNC, that the HPV vaccine can be given to individuals up to 45 years old, or that men can receive the HPV vaccine.

**Table 3:** Video Characteristics Stratified by Those That Mention HNC and Those That Do Not

Characteristic	Mentions HNC (n = 35) (n, %)	Does not mention HNC (n = 96) (n,%)	*P* value
Video metrics (mean, SD)			
Duration, minute	1.09 (0.89)	0.63 (0.62)	.007
Likes (#)	1787.74 (4031.38)	2506.23 (6608.69)	.455
Views (#)	62,744.11 (101,345.17)	70,602.76 (148,344.14)	.732
Comments (#)	98.17 (164.67)	86.04 (178.83)	.717
Account subscribers (#)	182,579.74 (472,537.31)	200,401.47 (393,054.09)	.843
Type of user account			
Patient	6 (17.14)	19 (19.79)	1.000
Physician	19 54.28571429	36 (37.50)	.109
Other providers	4 11.42857143	7 (7.29)	.481
Health care group	3 8.57142857	8 (8.33)	1.000
Health information	0 (0.00)	7 (7.29)	.189
Entertainment	3 8.57142857	10 (10.42)	1.000
Other	0 (0.00)	9 (9.38)	.111
Type of video			
Patient experience	3 (8.57)	8 (8.33)	1.000
Education	13 (37.14)	23 (23.96)	1.000
Advocacy	3 (8.57)	27 (28.13)	.020
Self‐promotion	3 (8.57)	5 (5.21)	.437
Entertainment	0 (0.00)	1 (1.04)	1.000
Other	1 (2.86)	7 (7.29)	.681
Country of origin			
United States	27 (77.14)	63 (65.63)	.210
Non‐United States	8 (22.86)	33 (34.38)	.288
Specialty			
Otolaryngology	2 (5.71)	0 (0.00)	.069
OBGYN	7 (20.00)	15 (15.63)	.599
Family medicine	3 (8.57)	15 (15.63)	.398
Other	9 (25.71)	11 (11.46)	.055
Mentioned can vaccinate up to 45	13 (37.14)	22 (22.68)	.119
Mentioned protects against cancer	‐	78 (80.41)	.003
Cervical cancer	30 (85.71)	59 (60.82)	.007
Vulva/vaginal cancer	10 (28.57)	5 (5.21)	.001
Penile cancer	14 (40.00)	5 (5.21)	<.001
Anal cancer	20 (57.14)	10 (10.31)	<.001
Head and neck cancer	‐	0 (0.00)	<.001
Cancer, not otherwise specified	3 (8.57)	11 (11.46)	.760
Mentioned men can receive vaccine	14 (40.00)	23 (23.96)	.080
DISCERN rating (mean, SD)			
Reliability	2.71 (0.41)	2.41 (0.53)	.001
Quality	2.13 (0.51)	1.71 (0.44)	<.001
Overall	2.91 (1.04)	2.03 (1.01)	<.001

Abbreviation: OBGYN, obstetrician‐gynecologist.

## Discussion

In this novel study, we report the characteristics of videos about HPV vaccination on a burgeoning social media platform and elucidate differences between videos involving physicians and mentioning HNC and those who do not. Our cohort of videos were generally of a low reliability and quality, with key topics such as vaccination age eligibility, the HPV vaccination's protective effect against HPV+ OPSCC, and male vaccination eligibility being infrequently mentioned. While physician‐involved videos were generally of higher quality, their mention of the aforementioned information was not significantly higher; otolaryngologists were also responsible for a small minority of these videos. However, videos mentioning HNC not only were significantly higher in quality and reliability but also had more significant mention of all HPV‐related cancers.

Unsurprisingly, social media's ubiquity in modern society involves health care. Digital dissemination of health information and patient education has been discussed throughout multiple specialties,^35^, ^36^ especially following mandatory social isolation policies instituted during the COVID‐19 pandemic.^37^ Otolaryngology patients have been found to use social media to seek medical advice and treatment decision‐making^38^; there has also been a report of increased pediatric tonsil stone presentation to otolaryngologic care secondary to a wide prevalence of patient‐made tonsil stone awareness videos on TikTok.^39^ Regarding physician use, content creators of various specialties continue to grow their digital presence across platforms,^40^, ^41^ leading to publications offering guidance regarding ethical physician social media use in specialties such as dermatology.^42^

Despite social media's high utility in health care as an affordable method of disseminating health information, it also has a well‐documented weakness of poor information quality due to its highly democratic and unregulated posting policies. TikTok has consistently been demonstrated in the literature to have a low quality and reliability of health‐related content over a plethora of topics,^28^, ^43^, ^44^, ^45^, ^46^, ^47^, ^48^ findings consistent with those of our own study. Furthermore, health‐related content on TikTok has also been found to be of significantly lower quality relative to that on YouTube.^28^, ^43^ While this is understandable given the platform's short‐form content optimized for mobile applications lending itself to attention‐grabbing content over more serious, informative videos that can be found on YouTube, it is simultaneously concerning given the platform's rise as a regular news source for many Americans. Most videos in our cohort were focused on HPV vaccination education or advocacy but still endorsed a low average overall DISCERN score of 2.29.

When stratifying our cohort by physician involvement, videos with physicians exhibited significantly higher reliability and quality scores, with an overall DISCERN score nearly approaching significance. We also found physicians to post significantly more educational content and significantly less patient experience or advocacy content relative to nonphysicians. Other studies of health‐related TikTok content have also found physicians post significantly higher quality content relative to non‐physicians, indicating they are correctly and appropriately using their medical training to educate the public.^43^, ^45^, ^46^, ^47^, ^48^

Still, there is undeniably a need for improvement in physician‐involved content given the relatively small absolute differences in DISCERN reliability, quality, and overall DISCERN scores between physicians and non‐physicians. Furthermore, there were no significant differences in cancer‐related content (vaccination up to 45 years, protective effect against different HPV‐related cancers, male vaccination eligibility) between physicians and non‐physicians despite the tremendous discrepancy in clinical training and knowledge. While previous work has investigated the prevalence of HPV cancer‐related content on TikTok, we are the first to report the frequency of information provided stratified by physician status. While we cannot ascertain the impact of certain factors such as physician knowledge, audience, and content‐creating ability on our results, the relatively low public knowledge of HPV and vaccination abilities suggest an acute need for physicians to use their public platforms to increase awareness of HPV+ OPSCC and encourage HPV vaccination.^16^

A somewhat surprising finding was how otolaryngologists were present in a minuscule proportion of all videos and physician‐involved videos despite the increasing emphasis on social media in the field; notably, both otolaryngologist‐featuring videos did mention HNC.^49^, ^50^ Although it is less surprising that OBGYNs were the most frequently represented specialty, HPV+ OPSCC's increased prevalence among HNCs and surpassing of cervical cancer as the most common HPV‐related cancer suggest a need for otolaryngologists to follow their OBGYN colleagues in raising awareness of HPV+ OPSCC and the importance of vaccination on social media to add addition expert validity and perspective to online discourse.^51^

When stratifying our video cohort by mention of HNC, those who mentioned HNC were longer in duration with significantly higher DISCERN reliability, quality, and overall scores; HNC‐including videos were significantly less likely to be advocacy‐focused but were significantly more likely to mention any, cervical, vulva/vaginal, penile, anal, and HNC. Furthermore, this subgroup had the highest reported overall DISCERN score. No one demographic is responsible for these higher quality videos. However, our findings indicate such videos exist and suggest HNC may only be mentioned in better researched, more comprehensive videos. As such, efforts to increase the quality of videos about HPV vaccination posted to TikTok may increase the quality of content or prevalence of HNC‐related information. However, HNC's mention in only a subset of better researched videos suggests its relation to HPV is not a primary point of discussion within this topic. Given the increasing incidence of HPV+ OPSCC, however, it may be beneficial to increase the prevalence of HNC in the HPV vaccination conversation to not only encourage HPV vaccination but also highlight other relevant risk factors such as tobacco and alcohol use.

Given the relatively lower rates of US HPV vaccine in the United States relative to that of other developed countries and increasing rates of HPV vaccine hesitation domestically, there remains a pressing need to increase HPV vaccination rate and awareness of the HPV vaccine's cancer‐preventing benefits.^52^, ^53^ As patients are unlikely to seek information from the medical literature often hidden beyond paywalls, increasing the quality and presence of content on social media websites such as TikTok—frequented by teenagers and young adults who are the prime demographic for HPV vaccination—remains an inexpensive and effective method of information dissemination. Otolaryngologists will be key contributors to this cause and should join OBYGNs in increasing awareness of HPV vaccination and use their expertise to lead the charge on HPV+ OPSCC awareness. Although the result of such efforts on HNC incidence may not be seen until as early as 2045, this long time to impact is precisely why we must start now.^54^

As with any study, ours is not without limitations. Although the DISCERN protocol has been validated and is used across specialties in evaluating social media posts, it remains an evaluative method not specific to otolaryngology. While the Instructional Videos in Otorhinolaryngology by Young Otolaryngologists of the International Federation of Otorhinolaryngological Societies‐grading system was recently introduced as a method of assessing otolaryngologic surgical videos based on consensus recommendations, it has not received wide adoption nor is validated.^55^ As such, the creation of a validated evaluative method for head and neck‐specific patient educational videos may provide more precise insight into the strengths and shortcomings of these patient‐facing materials. Further studies may also include understanding perceptions of HPV and vaccination as well as barriers to vaccination among young Americans. Understanding otolaryngologists' perceptions of social media use and its utility in patient education may also help identify methods of increasing otolaryngologic presence on social media, while developing expert consensus guidelines on the appropriate and ethical use of social media within our specialty may lend a guiding light to those interested in unsure of how to appropriate approach entering the digital space. Ultimately, efforts aimed at increasing the presence of otolaryngologists on social media can increase patient awareness and knowledge in an exceedingly digital world.

## Conclusion

We identified limitations in both quality, reliability, and content of the available content on TikTok related to HPV vaccination using the DISCERN grading system and our independent review. Given TikTok's rising status as a regular news source for many Americans, there is a need to not only increase the quality of content available but also to increase otolaryngologist involvement in raising public awareness of HPV vaccination and HPV+ OPSCC.

## Author Contributions

**Matthew E. Lin**, conception and design of work, data acquisition and analysis, interpretation of data, drafting of the manuscript, critical revision; **Oluwatobiloba Ayo‐Ajibola**, data acquisition and analysis, interpretation of data, drafting of manuscript, critical revision; **Carlos X. Castellanos**, data acquisition and analysis, interpretation of data, drafting of manuscript, critical revision; **Jonathan D. West**, Interpretation of data, drafting of manuscript, critical revision; **Neil Luu**, interpretation of data, drafting of manuscript, critical revision; **Niels C. Kokot**, conception and design of work, interpretation of data, critical revision.

## Disclosures

### Competing interests

None.

### Funding source

None.
